# Development and Evaluation of Molecular Diagnostic Tests for SARS-CoV-2 at English NHS Sites Throughout the COVID-19 Pandemic

**DOI:** 10.3390/v18050517

**Published:** 2026-04-30

**Authors:** Luke D. Griffith, Samir Dervisevic, Penny P. Powell

**Affiliations:** 1 Biomedical Research Centre, Norwich Medical School, University of East Anglia, Norwich Research Park, Norwich NR4 7TJ, UK; l.griffith@uea.ac.uk; 2 Eastern Pathology Alliance Department of Microbiology, Norfolk and Norwich University Hospital, Norwich NR4 7UY, UK; samir.dervisevic@nnuh.nhs.uk

**Keywords:** diagnostic assays, COVID-19, pandemic response, SARS-CoV-2, viral infection, disease detection, reverse-transcription PCR

## Abstract

The COVID-19 pandemic placed unprecedented pressure on diagnostic services worldwide. The first cases of severe acute respiratory syndrome coronavirus 2 (SARS-CoV-2) in the UK were confirmed on 31 January 2020, prompting National Health Service (NHS) laboratories to scale diagnostic procedures. The demand for testing rapidly exceeded historical norms for respiratory virus diagnostics, necessitating substantial government investment in consumables, assay development, and workforce expansion. This review presents a retrospective evaluation of SARS-CoV-2 diagnostic platforms deployed within the Norfolk and Norwich University Hospital (NNUH) trust and compares them with those implemented by other regional laboratories during the pandemic. It examines the molecular mechanisms, performance, scalability, and specificity of the multiple molecular testing approaches to optimise workflow based on the evolving technology. The integration of complementary platforms through a stratified testing strategy enabled high-throughput population screening while preserving diagnostic resolution for complex respiratory cases, substantially improving laboratory efficiency and resilience. The emerging diagnostic methodologies, RT-LAMP and CRISPR-based assays, are described, and we discuss their potential roles in future outbreaks. We critically evaluate the overall preparedness of UK health services for the COVID-19 pandemic and highlight key priorities for future pandemic preparedness at both local and national levels.

## 1. Introduction

In December 2019, patients in Wuhan, China, began presenting with infection caused by a previously unidentified coronavirus. The disease was characterised by symptoms consistent with pneumonia and, in severe cases, complications including respiratory failure and acute respiratory distress syndrome (ARDS) [1]. Subsequent analysis identified the causative agent as severe acute respiratory syndrome coronavirus 2 (SARS-CoV-2), with the associated disease designated coronavirus disease 2019 (COVID-19). Within one month of the first reported infections, SARS-CoV-2 spread to ten countries, prompting the World Health Organization (WHO) to declare a public health emergency of international concern (PHEIC) [1]. Global cases increased rapidly, rising from approximately 200,000 in March 2020 to 21 million by August of the same year [1]. This dramatic increase, alongside a global death toll of 761,799 during the same period, established SARS-CoV-2 as one of the most significant global pandemic viruses of the modern era [1].

The clinical impact of COVID-19 varies widely between individuals. Some infections remain asymptomatic, with approximately 17% or more of infected individuals showing no observable symptoms [2]. At the severe end of the disease spectrum, COVID-19 can progress to ARDS, characterised by systemic inflammation and multi-organ involvement affecting the lungs, heart, and gastrointestinal tract [3]. The combined effects of severe disease and rapid global spread resulted in substantial mortality. During the pandemic, case fatality rates varied considerably between regions, reaching a peak of 14.5% in Italy at one stage, while the estimated global average fatality rate was approximately 0.7% in June 2023 [4,5].

For the United Kingdom’s National Health Service (NHS), the COVID-19 pandemic presented significant operational challenges. Between 8 March 2020 and 8 April 2020, the UK experienced more than a 100-fold increase in daily confirmed COVID-19 cases, rising from 0.652 cases per million people in March to 66.706 cases per million in April [6]. To accommodate the large influx of patients requiring hospital care, NHS hospitals implemented extensive bed restructuring, increasing mechanical ventilation capacity by 53%, from a pre-pandemic baseline of 2711 beds to 4123 [7,8]. The need to expand mechanical ventilation capacity resulted in reductions of general and acute hospital beds, with an estimated 8.7% decrease in capacity, equivalent to approximately 8500 beds [7]. During this period, hospital occupancy patterns also shifted considerably. Public data indicate that between March and June 2020, 53,136 beds were occupied by non-COVID-19 patients, representing a 58% reduction compared to pre-pandemic levels recorded between January and March 2020 [8]. More broadly, NHS services were significantly disrupted during the pandemic, with substantial reductions observed in patient visits (42%), hospital admissions (28%), diagnostic procedures (31%), and therapeutic interventions (30%) [9]. Diagnostic services were particularly affected. NHS performance targets, specifically that diagnostic tests be completed within six weeks, declined from a 97% compliance rate to 42% between February and May 2020 [10]. To address the rapidly increasing burden of COVID-19, the NHS sought to expand SARS-CoV-2 diagnostic capacity.

Testing capacity in England was organised into two complementary streams: NHS hospital laboratories (Pillar 1), responsible for testing patients with clinical need; and a newly established network of high-throughput “Lighthouse Laboratories” (Pillar 2), designed to deliver mass community testing and based in Alderley Park (Manchester), Milton Keynes, Glasgow, and later Addenbrooke’s (Cambridge) [11]. This dual-track system enabled rapid expansion of testing capacity while preserving essential hospital laboratory services.

In March 2020, the NHS estimated that approximately 2000 molecular tests for SARS-CoV-2 could be performed daily [12]. By April 2020, this capacity had increased to 12,799 tests per day, and, by May 2020, the UK processing capacity was over 100,000 molecular tests daily, a 50-fold increase within just two months [12]. By October 2020, the UK had scaled testing capacity from minimal baseline levels to more than 2 million tests processed weekly [13]. NHS laboratories generally achieved turnaround times within 24 h, whereas Lighthouse Laboratories provided high-throughput processing of community samples. However, during peak demand periods, particularly in late 2020, turnaround times in Pillar 2 laboratories exceeded 90 h in some cases, largely due to logistical constraints in sample transport rather than analytical processing [14].

Rapid, accurate, and cost-effective detection methods for COVID-19 were essential throughout the pandemic to confirm cases, monitor disease spread, and track the evolution of the SARS-CoV-2 virus. Although rapid antibody-based detection methods, including lateral flow tests for home use, became increasingly prevalent as the pandemic progressed, the most clinically significant diagnostic approach remained molecular detection of viral RNA through various extraction and amplification techniques.

This review compares viral diagnostic technologies and the evolution of laboratory diagnostic processes implemented at a representative Pillar 1 NHS laboratory, the Norfolk and Norwich University Hospital (NNUH), during the pandemic. Specifically, it evaluates workflows involving magnetic bead-based nucleic acid extraction followed by two-step thermal multiplex tandem reverse-transcriptase real-time qPCR (MT-PCR), alongside an alternative platform using target capture, isothermal transcription-mediated amplification (TMA), and a dual kinetic assay (DKA) for SARS-CoV-2 detection. The review highlights the benefits and limitations of these platforms, examines how the NNUH laboratory adapted its testing procedures to optimise their use, and considers potential technological advancements, national-level initiatives, and alternative diagnostic methodologies that could further strengthen diagnostic responsiveness during future pandemics.

## 2. Initial SARS-CoV-2 Diagnosis: Primers and Qualitative Reverse-Transcriptase PCR Assay

Following the identification of SARS-CoV-2 as a major threat to public health and global economies, the international response was swift. In mid-January 2020, the WHO released the first official SARS-CoV-2 primers [15]. At NNUH, early diagnostic efforts for SARS-CoV-2 relied on these primers combined with nucleic acid extraction and manual reverse-transcriptase qualitative PCR (qRT-PCR) amplification. This followed a similar pattern to the Cambridge Microbiology Department, which saw initial diagnostic capacity established using a locally developed in-house qRT-PCR assay before transitioning to high-throughput platforms. Although these early protocols were low throughput and did not meet the diagnostic rigour typically expected under pre-pandemic conditions, they served as essential stopgap measures while commercial laboratories and industry partners developed automated, high-capacity testing platforms for widespread SARS-CoV-2 detection.

## 3. Two-Step Nucleic Acid Isolation and Thermal Multiplex Nested Reverse-Transcriptase Real-Time Qualitative PCR

**Figure 1:**
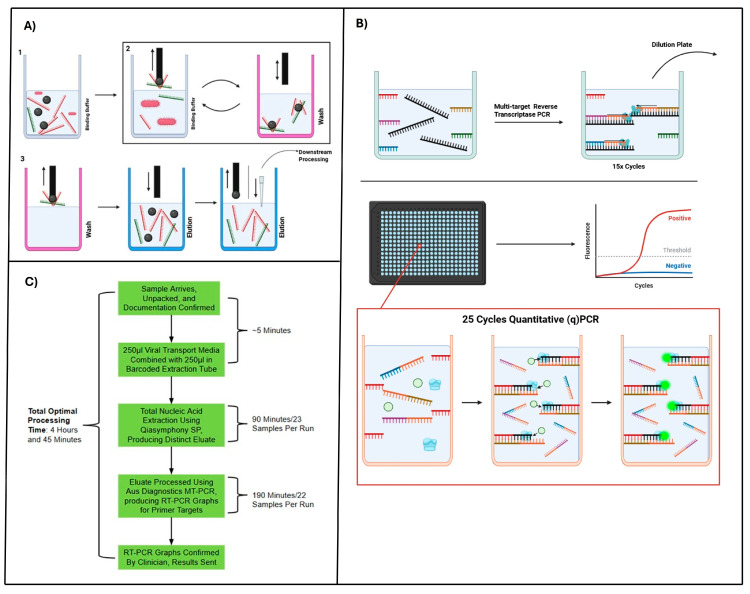
Initial-stage SARS-CoV-2 diagnostic workflow. (**A**) Nucleic acid extraction by the QIAsymphony SP. (1) Patient samples are combined with beads and extraction reagents. (2) Negatively charged nucleic acids are bound to positively charged magnetic beads and transferred through wash solutions. (3) Beads carrying nucleic acids are transferred into an elution buffer, where the nucleic acids are released into the solution; the beads are subsequently removed by the magnetic rod, yielding purified nucleic acids (created with BioRender). (**B**) The amplification and detection methodology of the Aus-Diagnostics MT-PCR platform. Nucleic acid eluates are amplified by multi-target reverse-transcriptase PCR for specific target pathogens and controls. The resulting cDNA is distributed into individual rows of a 384-well plate, with each column pre-coated with nested primers targeting a specific pathogen or control. A secondary quantitative PCR (25 cycles), performed in the presence of SYBR green, generates fluorescence signals proportional to the target amplification. Fluorescence is recorded for each well, producing output curves that are interpreted by clinicians for diagnostic decision-making (created with BioRender). (**C**) Overview of the complete first-stage COVID-19 sample processing workflow.

The initial automated COVID-19 diagnostic protocol implemented at the NNUH laboratory paired a nucleic acid isolation platform, the QIAsymphony SP, with amplification using the AusDiagnostics MT-PCR platform (Figure 1). The pipeline became feasible in March 2020, when a respiratory virus testing panel from AusDiagnostics, comprising assays for influenza A and B viruses (IAV/IBV), SARS-CoV-2, and respiratory syncytial virus (RSV), was approved by Public Health England (PHE) [16]. The QIAsymphony SP platform processed 23 patient samples in a single 90-minute run. With two instruments in operation, the laboratory achieved a maximum nucleic acid extraction rate of 31.5 patient samples per hour. The QIAsymphony SP automates the extraction of nucleic acid from multiple target pathogens across a broad variety of sample types, including viral transport medium (VTM), stool sample supernatant, and bronchoalveolar lavages [17]. The platform had seen significant use across diagnostic laboratories and was previously utilised by NNUH for the extraction of seasonal influenza virus nucleic acid in combination with the Qiagen DSP Virus//Pathogen Mini Kit^®^.

The QIAsymphony SP had been previously validated for extraction of IAV nucleic acid during the 2009 influenza pandemic, performing comparably to the previously validated bioMérieux NucliSENS easyMAG system across 50 clinical samples [18]. The QIAsymphony SP proved similarly effective for SARS-CoV-2, where it was widely utilised in multiple novel SARS-CoV-2 assay validations and variant-of-concern studies [19,20,21]. Following nucleic acid extraction, the AusDiagnostics MT-PCR platform could process 22 nucleic acid eluates in approximately 190 min. Using three MT-PCR instruments, in conjunction with the QIAsymphony SP platforms, the laboratory achieved a throughput of approximately 20.8 patient samples per hour.

The diagnostic workflow began with the combination of 250 µL of nasopharyngeal VTM with 250 µL of carrier RNA, which enhances nucleic acid recovery during extraction [22]. Nucleic acid extraction was performed using the Qiagen DSP Virus/Pathogen Mini Kit^®^, enabling the isolation of both host and pathogen genetic material. The samples were loaded into sample preparation cartridges and lysed with proteinase K solution^®^ to release the nucleic acids. Following lysis, a binding buffer and positively charged magnetic beads were added to the solution. These beads selectively bind negatively charged nucleic acids, enabling their separation. After incubation, the magnetic bead–nucleic acid complexes were transferred to a new cartridge containing a wash solution using a magnetic rod. The extraction and wash steps were repeated multiple times to maximise nucleic acid yield and purity. Finally, the beads and bound nucleic acids were transferred to an elution buffer^®^ to release the nucleic acids, producing a purified eluate for downstream processing using the Aus Diagnostic MT-PCR platform (Figure 1A).

The SARS-CoV-2, IAV/IBV, and RSV 8-well assay kit was used for all COVID-19 samples using the Aus Diagnostics MT-PCR platform (Figure 1B). A master mix containing DNA polymerase, reverse transcriptase (RT), oil, water, and an internal assay control denoted SPIKE was combined with individual extracted nucleic acid eluates. The resulting solution was then transferred to an individual assay well containing step-one control and pathogen-specific primers for the testing panel. Following the transfer, a multiplex RT-PCR (15 cycles) was performed to generate complementary DNA (cDNA), amplifying both control and pathogen targets if present within each clinical sample. The resulting cDNA was transferred to a 384-well dilution plate and combined with SYBR green, a non-specific DNA intercalating dye, and step-two nested PCR primers specific to the assay targets and controls for second-stage real-time qPCR. This nested amplification protocol enhances both the sensitivity and specificity of the assay, ensuring that only samples containing the correct primer targets undergo efficient amplification. Each of the 16 rows within the dilution plate contains primers for a single assay, ensuring that each diagnostic target is tested independently.

During amplification, newly synthesised double-stranded DNA incorporated SYBR green dye, generating a fluorescent signal proportional to the quantity of target nucleic acid present in each well. The second-stage real-time PCR was performed over 25 cycles, with fluorescence intensity recorded at each cycle. A positive result was defined by fluorescence exceeding a predefined threshold between cycles 10 and 20 of the 25 total cycles. Assays were considered invalid if no signal was detected from either the SPIKE control or the human reference control (NONO). All flagged positive results were subsequently reviewed through visual inspection of amplification curves by clinicians prior to reporting, minimising the risk of false-positive results.

The AusDiagnostics multi-viral panel used in this early workflow allowed for the simultaneous detection of multiple common respiratory viruses. Although this respiratory virus panel provided extensive diagnostic coverage, it also increased the associated costs. The cost–benefit balance of the pipeline was further affected by the source of the sample pool, which included a substantial proportion of surveillance testing, admission screenings, and asymptomatic monitoring. While beneficial for conventional respiratory virus detection, the full diagnostic depth of the panel was often unnecessary for SARS-CoV-2 screening, adding extra costs without improving clinical utility. At East Suffolk and North Essex NHS Foundation (Ipswich and Colchester), SARS-CoV-2 detection was performed using the similar Roche Cobas 5800 system. Although AusDiagnostics platforms were also available, the bulk of testing was not performed utilising this technology at this site due to the laboratory use of Roche reagents.

The theoretical optimal processing time for this early SARS-CoV-2 diagnostic pipeline was approximately 4 h and 45 min per sample (Figure 1C). However, the relatively low throughput of the combined platforms, 46 patient samples per 90 min for the two QIAsymphony SP instruments and 66 patient samples per 190 min for the three AusDiagnostics MT-PCR systems, limited the overall capacity. Consequently, the workflow was often insufficient to meet the volume of incoming samples, leading to backlogs and delays in result reporting. Despite these limitations, an automated pipeline represented a substantial improvement over manual methods, increasing sample throughput while maintaining high diagnostic accuracy.

## 4. Target Capture, Isothermal Amplification, and Dual Kinetic Assay

The increasing volume of diagnostic samples, high reagent costs, and slow processing times of the original pipeline increased the need for alternative testing methods to ensure efficient sample turnover. To address these limitations, the Aptima SARS-CoV-2 assay, performed on the Hologic Panther platform, was incorporated into the NNUH COVID-19 diagnostic workflow. This system provided a high-throughput alternative to the thermal multiplex MT-PCR pipeline. This adoption followed a similar pattern to both Addenbrooke’s Hospital (Cambridge) and East Suffolk and North Essex NHS Foundation Trust, which transitioned away from a lower throughput in-house assay and automated qualitative PCR platform, the Roche Cobas 500, respectively, to Panther systems to improve sample turnover.

**Figure 2:**
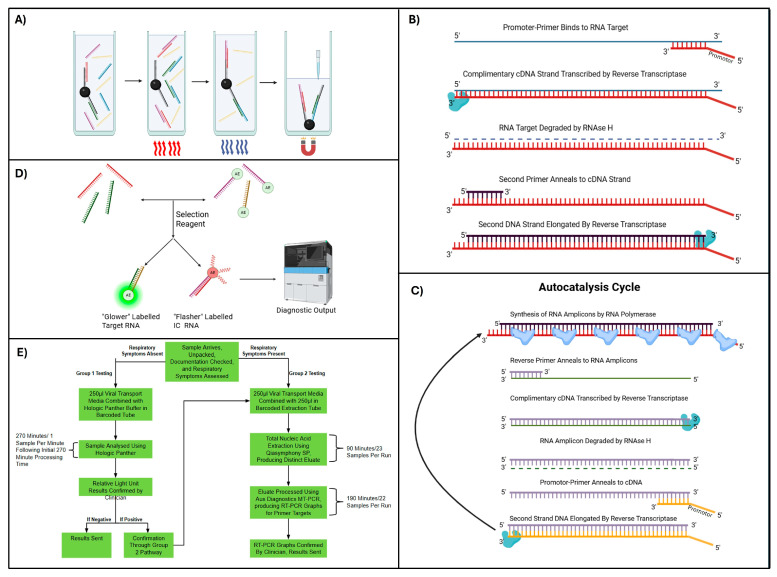
Hologic Panther SARS-CoV-2 diagnostic workflow. (**A**) Target capture. Samples are incubated with magnetic beads coated with poly-deoxythymidine anchors and complementary poly-deoxyadenosine capture probes. On heating, the probes hybridise to target nucleic acids; cooling re-associates complexes with the beads, allowing for magnetic separation and wash steps (created with BioRender). (**B**) First-stage transcription-mediated amplification (TMA). Target RNA is reverse transcribed to complementary DNA (cDNA). RNase H degrades the RNA template, and second-strand synthesis generates double-stranded cDNA containing a promoter sequence (created with BioRender). (**C**) Autocatalytic amplification begins with T7 RNA polymerase producing RNA amplicons from double-stranded cDNA templates. These are reverse transcribed and converted back into double-stranded DNA, enabling continuous isothermal amplification (created with BioRender). (**D**) Dual Kinetic Assay. Dual kinetic assay (DKA) detection. Amplicons hybridise with acrylamide ester-labelled probes specific to the targets and internal control (IC). Unbound probes are removed, and chemiluminescence is measured in relative light units (RLUs) for result interpretation (created with BioRender). (**E**) Overview of the second-stage NNUH COVID-19 workflow incorporating Type 1 and Type 2 sample classification.

The Hologic Panther platform uses TMA, combined with target capture and DKA detection, to identify two conserved regions within the SARS-CoV-2 ORF1ab gene. The assay includes an internal control (IC), consisting of a synthetic oligomer processed alongside the target viral RNA to monitor performance. Positive and negative controls are included with each reagent kit, which supports up to 250 tests, allowing for a maximum of 248 patient samples once controls are accounted for. For processing, 500 µL of patient nasopharyngeal VTM is added to a barcoded RespDirect™ Collection Kit containing lysis buffer, then mixed with the target capture reagent (TCR). The TCR contains magnetic microparticles coated with deoxythymidine capture oligomers complementary to the target RNA sequence, anchored to the microparticles via deoxyadenosine residues in a heat-dependent manner (Figure 2A). Heating uncouples the deoxythymidine residues, enabling hybridisation with target RNA. Subsequent cooling re-couples the oligomers to the microparticles, immobilising captured RNA and IC molecules. The magnetic microparticles are retained during aspiration and wash steps to remove unbound material. TMA enriches target sequences through a two-stage amplification process. In the first stage, a promoter-primer containing a 5′ T7 RNA polymerase promoter binds to the 3′ end of the target RNA (Figure 2B). RT synthesises a cDNA strand incorporating the promoter sequence as its intrinsic RNase H activity degrades the original RNA template. Secondary primers bind to the cDNA 3′ region, allowing RT to generate double-stranded DNA (dsDNA) [23,24,25]. In the second stage, T7 RNA polymerase binds the embedded promoter, forming a transcription complex that transcribes dsDNA into multiple RNA amplicons (Figure 2C). These RNA amplicons then serve as templates for further reverse transcription and T7 promoter-primer binding, resulting in autocatalytic exponential amplification of target sequences [23,24,25]. When compared with conventional RT-PCR, TMA offers several advantages. TMA does not require thermal cycling due to its isothermal capacity, allowing for faster processing, and studies suggest it may achieve higher overall sensitivity [26,27].

The final detection step is the DKA (Figure 2D). RNA amplicons are hybridised with single-stranded chemiluminescent DNA probes labelled with acridinium ester (AE), complementary to either the target RNA or IC. Non-hybridised probes are removed by a selection reagent^®^, and the emitted light is measured in relative light units (RLUs) using a luminometer. The assay differentiates IC and viral RNA signals based on kinetic profiles: the IC probe produces a rapid “flasher” signal, while the SARS-CoV-2 probe generates a slower “glower” signal. Positive results are determined using RLU thresholds combined with a kinetic curve type, with flagged samples displayed on the user interface. All RLU measurements for positive samples are visually inspected by clinicians before release to identify any potential assay aberrations.

The Aptima SARS-CoV-2 workflow achieved a diagnostic output of 60 samples per hour following an initial 270-min processing window. This high-throughput capability allowed most routine SARS-CoV-2 testing at the NNUH laboratory to transition from the QIAsymphony SP/MT-PCR workflow to the Hologic Panther. However, the initial 4.5-h processing time remained limiting for urgent cases requiring rapid turnaround. Additionally, the assay’s design restricts detection to SARS-CoV-2 RNA only, meaning that complex respiratory infections still required the broader MT-PCR workflow.

A further limitation of the Aptima assay was the frequency of control failures. Each 250-test reagent kit was validated with commercial controls, and failure of a control rendered all patient samples processed in the subsequent 270-min window invalid, necessitating immediate retesting. This could result in up to 248 results being delayed or invalidated, creating significant backlogs. Despite these drawbacks, the Hologic Panther remained an essential component of NNUH’s SARS-CoV-2 testing portfolio, providing automated high-throughput screening at a reduced cost relative to the original MT-PCR pipeline.

## 5. Integrating Platforms for Optimal Diagnostic Output

Following the introduction of a high-throughput screening platform, the NNUH laboratory adapted its testing procedures to optimise the use of available diagnostic platforms (Table 1). In the revised workflow (Figure 2E), patient samples were stratified into two distinct groups. The first group comprised patients presenting with non-specific COVID-19 symptoms, those requiring hospital admission, and individuals undergoing routine NHS screening.

These samples were processed using the high-throughput Hologic Panther platform, maximising processing capacity despite its limited diagnostic breadth. All positive results were subsequently re-analysed using the QIAsymphony SP/AusDiagnostics MT-PCR workflow using a viral transport medium from the original nasopharyngeal swab. This secondary testing step ensured result accuracy and reduced the risk of false positives associated with the Aptima SARS-CoV-2 test.

The second group included patients with clearly defined respiratory symptoms documented in clinical records. These samples were analysed exclusively using the QIAsymphony SP/AusDiagnostics MT-PCR workflow, enabling the simultaneous detection of SARS-CoV-2, influenza virus A/B, and RSV, thereby providing greater diagnostic resolution. This stratified strategy, reserving high-throughput platforms for routine screening and targeted platforms for complex diagnostics, substantially improved laboratory capacity and turnaround times while optimising cost-effectiveness. Nevertheless, future evaluation of platform sensitivity, specificity, and reagent utilisation is essential. Further integration of advanced multiplexed or high-throughput technologies will enhance both throughput and diagnostic precision, providing an economically sustainable framework for pandemic-scale testing. The following sections explore emerging innovations and their potential use in pandemic response.

## 6. Point-of-Care Multiplex Testing

Point-of-care (POC) testing platforms aim to deliver rapid diagnostic results within the clinical setting, bypassing the need for samples to be processed through conventional laboratory workflows. The Roche Cobas Liat is a rapid, multiplex POC test initially designed to detect IAV/IBV and RSV. During the COVID-19 pandemic, its diagnostic panel was expanded to include SARS-CoV-2. Each cartridge contains all of the necessary reagents for nucleic acid extraction and RT-qPCR. Individual reagents are separated by heat-sensitive seals that are broken to initiate controlled reagent mixing.

In practice, a nasopharyngeal swab in VTM is loaded into the cartridge. The total nucleic acids are extracted using silica-coated magnetic beads, eluted, and subjected to RT-qPCR. Unlike conventional thermal cycling in stationary microplates, the Cobas Liat uses microfluidic channels to shuttle the sample between heated segments, markedly reducing amplification time. This enables diagnostic results within approximately 20 min, making the platform particularly useful for urgent clinical decisions, such as organ donor screening or patient triage. Alongside the Cobas Liat, the NHS also utilised other POC testing platforms, including the SAMBA II and Cepheid Xpert Xpress assays, as part of their diagnostic approach.

## 7. Reverse-Transcription Loop-Mediated Isothermal Amplification (RT-LAMP)

Alternative amplification methods emerged during the COVID-19 pandemic to mitigate reagent shortages and enable high-throughput screening, reserving limited laboratory resources for cases requiring detailed analysis. Reverse-transcription loop-mediated isothermal amplification (RT-LAMP) is a widely used nucleic acid amplification technique, previously applied to pathogens such as the West Nile virus and SARS, as well as veterinary and plant pathogens [28,29].

RT-LAMP employs four to six primers targeting distinct regions of the assay gene: a forward inner primer (FIP), a forward outer primer (FOP), a backward inner primer (BIP), and a backward outer primer (BOP). The inner primers contain sequences complementary to both sense and antisense regions of the target cDNA, linked by a poly-thymidine spacer [30,31]. Optional loop primers can be added to further enhance the reaction speed and sensitivity [30]. A DNA polymerase with strong strand displacement activity, typically Bst DNA polymerase, drives amplification [31].

The assay is performed at a constant 65 °C, allowing heat-stable reverse transcriptase to convert RNA to cDNA. Partial denaturation at this temperature enables the inner primers to initiate strand displacement, while outer primers generate complementary single-stranded DNA with loop structures at both ends, forming a “dumbbell” configuration. These structures quickly convert into stem-loop DNA, which serves as a template for inner and loop primers, supporting exponential amplification of target sequences [31]. The resulting amplified product is compatible with multiple detection methodologies, including both fluorometric and colorimetric techniques.

## 8. CRISPR-Cas-Based Diagnostics

CRISPR-Cas systems, originally characterised as prokaryotic adaptive immune mechanisms, are increasingly being repurposed for nucleic acid diagnostics [32,33]. In nature, CRISPR RNA (crRNA) guides Cas proteins to cleave foreign DNA or RNA, protecting the host from invading genetic material [33]. In diagnostic applications, synthetic guide RNAs (gRNAs) are designed to target specific DNA or RNA sequences, directing Cas-mediated cleavage at these sites [33]. Detection exploits the enzymatic properties of Cas proteins: Cas12, which targets DNA, exhibits indiscriminate single-stranded DNA (ssDNA) trans-cleavage after target binding, while Cas13, which targets RNA, cleaves single-stranded RNA (ssRNA) reporters [33,34]. By incorporating fluorescent ssDNA or ssRNA probes, cleavage events generate measurable signals corresponding to the presence of target nucleic acids.

During the COVID-19 pandemic, CRISPR-Cas assays were explored as alternatives to conventional RT-qPCR. The SARS-CoV-2 DNA endonuclease-targeted CRISPR trans reporter (DETECTR) assay, developed in 2020, combined RT-LAMP amplification with Cas12-mediated detection of SARS-CoV-2 sequences [35]. The assay targets the envelope (E) and nucleoprotein (N) genes, alongside the human RNase P gene as an internal control. Following RT-LAMP, Cas12 is directed to amplified targets by specific gRNAs, inducing indiscriminate cleavage of FAM-biotin reporter molecules. Cleavage is visualised on a lateral flow strip, with the control line detecting uncleaved probes and the test line indicating cleaved probes [35].

## 9. Comparison of Detection Methods for SARS-CoV-2

This review examined a range of diagnostic methods for the detection of SARS-CoV-2 from patient samples. The following section provides a comparative evaluation of the technical performance of assays implemented at the NNUH laboratory along with alternative diagnostic platforms, integrating with the published performance data to provide a direct comparison of the methods (Table 2). Whereas published comparative studies across all of the described platforms and assays remain limited, reported performance metrics have been synthesised to provide an assessment of their relative clinical utility.

Across molecular diagnostic testing, RT-PCR-based assays consistently demonstrate the highest diagnostic accuracy, with reported sensitivities typically ranging from 93 to 100% and specificities exceeding 98% in most studies [36]. Within the NNUH laboratory, the AusDiagnostics MT-PCR platform represented the most accurate and consistent diagnostic pipeline. Published validation data reported a specificity of 98.3% for the Aus diagnostic testing panel [37]. In a cohort of 7839 samples, SARS-CoV-2 was detected in 127 cases, with 92.9% initial agreement with an in-house TaqMan assay [37]. Resolution of discordant results through repeat testing and sequencing increased overall agreement, yielding an estimated diagnostic accuracy of approximately 98.4% [37].

In contrast, the Aptima SARS-CoV-2 TMA assay demonstrates more variable performance across studies. Several evaluations reported high overall agreement with RT-PCR-based assays, ranging from 94.7 to 98.9% [38,39,40,41,42]. However, it is important to note that such studies often rely on RT-PCR-based pre-selected positive cohorts, introducing selection bias that may overestimate Aptima assay sensitivity [43]. For example, in a study of 500 Aptima-positive samples, approximately 15.2% were not confirmed by subsequent RT-PCR analysis, suggesting a potential for false-positive results [43]. This may potentially be the result of low viral load samples, with a similar false-positive rate of 11.9% observed in high CT samples [41]. Overall, the available evidence suggests that while the Aptima TMA assay offers high throughput and operational efficiency, its performance may be more variable than conventional RT-PCR, therefore requiring confirmatory testing.

**Table 2:** Comparison of diagnostic assays and platforms for SARS-CoV-2.

Assay/Platform	Methodology	Sensitivity (%)	Specificity (%)	Turnaround Time	Advantages	Limitations
AusDiagnostics MT-PCR	Multiplex tandem RT-PCR	98.4% [37]	~98.3% [37]	4–6 h	High diagnostic accuracy; multiplex detection; robust performance	Lower throughput compared to high-throughput systems; requires laboratory infrastructure
Aptima SARS-CoV-2	Transcription-mediated amplification (TMA)	84.8–98.9% [38,39,40,41,42,43]	96.1–100% [38,39,40,41,42]	4 h 30 min	High throughput; automated workflow	False positives reported (~15.2%) [42]; requires confirmatory testing
Roche Cobas Liat	Rapid RT-PCR (POCT)	90.4–100% [44,45]	98.7–99% [40] Reported false-positive rate of 9.6% [45]	20 min	Rapid results; minimal training; point-of-care testing	Low throughput; potential high false-positive rate [45]; requires confirmatory lab testing
OptiGene RT-LAMP (RNA)	Isothermal amplification (RT-LAMP)	95% [46]	99–100% [46]	30–60 min	High specificity; faster than RT-PCR; less reagent dependence	Reduced sensitivity vs. RT-PCR in some contexts; requires RNA extraction
OptiGene Direct RT-LAMP	Direct RT-LAMP	79% [46]	98–100% [46]	30 min	Non-invasive sampling; rapid; scalable screening	Reduced sensitivity in low viral load
DETECTR (CRISPR-Cas12)	CRISPR-based detection	95% [35]	100% [35]	30–40 min	Rapid; high specificity; reduced reagent dependency	Limited large-scale validation; regulatory uncertainty; not widely implemented

A somewhat different approach to testing for SARS-CoV-2 was adopted at Addenbrooke’s Hospital in Cambridge. Point-of-care (POC) testing was implemented very early into the pandemic to support rapid clinical decision-making, while NNUH in Norwich did not use this technology as part of its routine diagnostic approach. POC RT-PCR platforms, such as the Roche Cobas Liat, provide rapid diagnostic insight with minimal operator training. These systems are particularly valuable in acute clinical settings where rapid decision-making is required. Overall, the SARS-CoV-2 Liat assay showed high sensitivity (96.7–100%) and specificity (98.7–99.9%) from a meta-analysis of existing performance evaluations [44]. However, the single study that did not report specificity was excluded. Notably, this study reported a substantially elevated false-positive rate of approximately 9.6% [45]. This observation was supported by a 2021 Food and Drug Administration (USA) alert that warned of false positives associated with the Liat assay [45]. Given the risk of elevated false-positive results, confirmatory testing again would be required if the Liat system were to be implemented at NNUH. Collectively, these findings highlight the inherent trade-offs between speed, throughput, and diagnostic accuracy in point-of-care testing platforms. While the Liat system offers significant clinical utility in scenarios requiring rapid diagnostic insight, it is best deployed as part of an integrated point-of-care testing strategy within broader laboratory workflows rather than as a standalone diagnostic solution.

Alternative amplification methodologies, including RT-LAMP and CRISPR-based diagnostics, offer distinct advantages but demonstrate greater variability in performance. RT-LAMP offers several advantages over RT-qPCR. Its isothermal amplification significantly reduces processing time and removes the need for thermocyclers, lowering both equipment and reagent costs. Additionally, the use of multiple primers enhances specificity compared with conventional RT-qPCR [31]. The OptiGene RT-LAMP assay, evaluated across multiple NHS sites, achieved a sensitivity of approximately 91–97% and a specificity of 99–100% when applied to extracted RNA samples [46]. However, performance declined when applied to direct raw sample testing, with an overall accuracy of approximately 79%, reflecting reduced sensitivity in low viral load samples [46]. This variability is consistent with broader observations that RT-LAMP assays perform optimally in high viral load contexts but may be less reliable for screening early-stage infections [47,48].

CRISPR-based diagnostics, such as the DETECTR platform, represent an emerging alternative with promising analytical performance. Evaluation of DETECTR using 83 respiratory swab samples demonstrated 95% positive predictive agreement and 100% negative predictive agreement compared to RT-qPCR [35]. The assay can be completed in approximately 30–40 min and reduces dependence on conventional PCR reagents [35]. Despite these advantages, limited large-scale validation and evolving regulatory status have restricted widespread clinical adoption. For instance, although DETECTR previously received Emergency Use Authorization, this was later withdrawn [49].

Taken together, these findings highlight that while RT-PCR-based platforms remain the gold standard for diagnostic accuracy, alternative methodologies provide important complementary roles depending on the clinical context. High-throughput systems, such as the Hologic Panther Aptima assay, offer scalability for mass testing, point-of-care platforms, such as Cobas Liat, enable rapid clinical decision-making, and emerging technologies, such as RT-LAMP and CRISPR, expand diagnostic capacity under resource constraints. The NNUH experience reflects these broader trends, demonstrating that optimal diagnostic strategies rely not on a single platform but on the integration of multiple technologies tailored to clinical and operational requirements.

## 10. Discussion and Future Directions

The SARS-CoV-2 pandemic highlighted both the strengths and structural limitations of diagnostic strategies implemented across NHS England. Central coordination enabled the rapid mobilisation and scaling of diagnostic capacity, supporting both clinical management and population-level surveillance. Within NNUH, this was mirrored by the rapid transition from manual qRT-PCR workflows to automated and high-throughput platforms, demonstrating the adaptability of NHS laboratory services under extreme pressure. However, several systemic and operational challenges limited the overall effectiveness of the response.

One of the principal national-level limitations was the fragmentation between Pillar 1 NHS laboratories and Pillar 2 Lighthouse Laboratories. Although the creation of the Lighthouse network enabled the rapid expansion of testing capacity, the separation of these systems resulted in limited interoperability, delayed data integration, and reduced clinical feedback to requesting healthcare providers. Greater integration between these pillars, particularly through shared laboratory information systems and unified reporting pipelines, could have improved turnaround times, data accessibility, and clinical utility [50]. In contrast, the NNUH experience demonstrated the benefits of integrated workflows at a local level, where stratified testing approaches, combining high-throughput screening with confirmatory multiplex diagnostics, optimised turnaround times, diagnostic accuracy, and cost-effectiveness. This contrast highlights the importance of unified diagnostic networks across both national and local systems.

Logistical constraints also emerged as a major bottleneck. While analytical throughput within centralised facilities, such as Lighthouse Laboratories, was high, delays in sample transport and processing contributed to prolonged turnaround times during peak demand [11]. At NNUH, similar pressures were observed in the early MT-PCR workflow, where limited throughput and extended processing times led to backlogs despite automation. The later implementation of the Hologic Panther platform improved throughput substantially but highlighted the compromise between speed and diagnostic breadth. These findings support the need for a more decentralised diagnostic model, incorporating regional high-throughput hubs alongside strengthened local laboratory capacity to improve responsiveness to regional surges in demand [13].

The pandemic further exposed limitations in diagnostic preparedness. Early in 2020, laboratory capacity was constrained by shortages in trained personnel, limited instrumentation, and an inability to scale rapidly [15]. This was consistent with the initial reliance on manual workflows at NNUH, which served as essential stopgap measures but lacked the scalability required for pandemic-level testing. Workforce limitations were a key contributing factor, as the rapid escalation in testing demand outpaced the availability of trained laboratory personnel, necessitating redeployment and accelerated training programmes. Future preparedness strategies should prioritise the development of a flexible, cross-trained diagnostic workforce and establish reserve staffing frameworks that can be rapidly activated during public health emergencies.

In addition, heavy reliance on closed, proprietary diagnostic platforms created vulnerabilities in the global supply chain. Shortages of reagents and consumables significantly constrained testing capacity during the early stages of the pandemic [11]. This was reflected both nationally and locally, where platform selection and workflow optimisation were influenced not only by assay performance but also by reagent availability. Diversification of suppliers, investment in open-platform technologies, and the development of validated contingency assays, including RT-LAMP and CRISPR-based assays, would enhance resilience against future disruptions [11].

Despite these challenges, several aspects of the testing strategy were notably effective. National coordination facilitated rapid procurement and deployment of RT-qPCR technologies, while collaboration between public institutions, academia, and industry enabled large-scale diagnostic innovation. At the local level, NNUH demonstrated how integration of complementary diagnostic platforms could optimise throughput, cost-efficiency, and diagnostic accuracy. Furthermore, integration with genomic surveillance initiatives, particularly through the COVID-19 Genomics UK (COG-UK) Consortium, enabled large-scale sequencing and identification of emerging variants, strengthening epidemiological monitoring and public health response.

However, despite advances in genomic surveillance, NHS clinical management has generally not incorporated strain-specific differences into treatment strategies. Evidence from other respiratory pathogens, such as the influenzavirus, demonstrates the potential clinical value of viral subtyping, with certain strains associated with increased disease severity, intensive care admission, and mortality risk [51,52,53]. Expanding subtyping capabilities within NHS diagnostic workflows would, therefore, provide important prognostic insight, enabling the earlier identification of high-risk patients and more targeted clinical management.

Beyond pathogen characterisation, integrating personalised medicine approaches into diagnostic workflows represents an additional opportunity to enhance patient care. Host genetic variation, including single-nucleotide polymorphisms (SNPs), has been shown to influence susceptibility to severe COVID-19 and other respiratory infections [54,55,56,57,58]. In future, residual nucleic acid extracted during routine diagnostic workflows can be used to identify such markers, enabling risk stratification and more tailored clinical interventions.

Taken together, these findings highlight that optimal diagnostic strategies rely not on a single platform, but on the integration of multiple complementary technologies and analytical techniques. High-throughput systems such as transcription-mediated amplification enable large-scale screening, while multiplex RT-PCR platforms provide essential diagnostic resolution for complex infections. Emerging methodologies, including RT-LAMP and CRISPR-based diagnostics, offer additional flexibility and may mitigate reagent dependency during future outbreaks. Finally, incorporation of new analytical methodologies, including virus subtyping and SNP detection, has the potential to improve patient outcomes while optimising healthcare resource allocation.

Looking forward, key priorities for improving pandemic preparedness include the following: (1) the establishment of fully integrated diagnostic networks bridging clinical and community testing; (2) investment in scalable, decentralised laboratory infrastructure; (3) development of robust and diversified supply chain strategies; and (4) sustained funding for workforce training and retention. Embedding these principles within national preparedness frameworks will be essential to ensuring a more resilient and responsive diagnostic system in future public health emergencies. Additionally, maintaining surge diagnostic capacity, adopting flexible and open testing platforms, and strengthening collaboration between public and private sectors will be critical to improving future pandemic responses.

## Figures and Tables

**Table 1:** Summary of NNUH COVID-19 diagnostic workflows.

Platform	Experimental Process	Diagnostic Range	Sample Throughput
Manual qRT-PCR (Early 2020)	Manual nucleic acid extraction from nasopharyngeal VTM, followed by qualitative RT-PCR using WHO primers	SARS-CoV-2 only	Low; limited by manual extraction and amplification steps
QIAsymphony SP/AusDiagnostics MT-PCR	Automated nucleic acid extraction (QIAsymphony SP), followed by multiplex RT-PCR with nested amplification (AusDiagnostics MT-PCR)	SARS-CoV-2, influenza virus A/B, RSV	Moderate; 31.5 samples/h (QIAsymphony SP), 20.8 samples/h (MT-PCR)
Hologic Panther/Aptima SARS-CoV-2	Isothermal transcription-mediated amplification (TMA) with target capture and dual kinetic assay (DKA) detection	SARS-CoV-2 only	High; 1 sample per minute
Integrated Workflow (Stratified Approach)	Sample triage into Panther for routine/high-volume screening, MT-PCR for positive confirmation or complex respiratory cases	Panther: SARS-CoV-2; MT-PCR: SARS-CoV-2, influenza A/B, RSV	Combined throughput optimised for lab capacity

## Data Availability

No new data were created or analyzed in this study.

## Abbreviations

The following abbreviations are used in this manuscript:

SARS-CoV-2	Severe Acute Respiratory Syndrome Coronavirus 2
NHS	National Health Service
NNUH	Norfolk and Norwich University Hospital
ARDS	Acute Respiratory Distress Syndrome
COVID-19	Coronavirus Disease 2019
PHEIC	Public Health Emergency of International Concern
WHO	World Health Organisation
MT-PCR	Multiplex Tandem Reverse-Transcriptase Real-Time qPCR
TMA	Transcription-Mediated Amplification
DKA	Dual Kinetic Assay
qRT-PCR	Qualitative Reverse-Transcriptase PCR
RSV	Respiratory Syncytial Virus
PHE	Public Health England
IAV	Influenza A Virus
IBV	Influenza B Virus
VTM	Viral Transport Media
RT	Reverse Transcriptase
cDNA	Complimentary DNA
IC	Internal Control
dsDNA	Double-Stranded DNA
TCR	Target Capture Reagent
AE	Acridinium Ester
RLU	Relative Light Unit
POC	Point of Care
RT-LAMP	Reverse-Transcription Loop-Mediated Isothermal Amplification
SARS	Severe Acute Respiratory Virus
ssDNA	Single-Stranded DNA
ssRNA	Single-Stranded RNA
crRNA	CRISPR RNA
gRNA	Guide RNA
DETECTR	DNA Endonuclease-Targeted CRISPR Trans Reporter
E	Envelope
N	Nucleoprotein
COG-UK	COVID-19 Genomics UK Consortium
